# Synthesis and characterization of the cyanobenzene-ethylenedithio-TTF donor

**DOI:** 10.3762/bjoc.11.106

**Published:** 2015-06-03

**Authors:** Sandrina Oliveira, Dulce Belo, Isabel Cordeiro Santos, Sandra Rabaça, Manuel Almeida

**Affiliations:** 1 C²TN, Instituto Superior Técnico, Universidade de Lisboa, Estrada Nacional 10, P-2695-066 Bobadela LRS, Portugal

**Keywords:** cross-coupling, cyanobenzene, cyclic voltammetry, dissymmetric tetrathiafulvalene, electro-active donors

## Abstract

A dissymmetric TTF-type electron donor, cyanobenzene-ethylenedithio-tetrathiafulvalene (CNB-EDT-TTF), was obtained in high yield, by a cross-coupling reaction with triethyl phosphite between 2-thioxobenzo[*d*][1,3]dithiole-5-carbonitrile and 5,6-dihydro-[1,3]dithiolo[4,5-*b*][1,4]dithiin-2-one. This new donor was characterized namely by single crystal X-ray diffraction, cyclic voltammetry, NMR, UV-visible and IR spectroscopy.

## Introduction

The tetrathiafulvalene molecule (TTF) and its many derivatives, due to its unique π-donor properties, have been at the basis of the large majority of organic metals and superconductors known so far [1–2]. Their success as building blocks for conducting materials is due to unique π-donor properties of TTF with two oxidized states readily accessible and the possibility of large intermolecular interactions in solid state through rather extended π-orbitals of these flat molecules. The exploration of new TTF derivatives in this context has followed two main guidelines. First, the further extension of the conjugated π-system to render more accessible the different oxidation states, and maximize the intermolecular interactions between planar molecules that tend to be organized in the solid state as stacks or layers with their long axis parallel to each other. Second, the incorporation of additional sulfur and other chalcogen atoms in the molecular periphery to promote side intermolecular interactions along the molecular plane allows a possible 2D or 3D character to the electronic interactions.

More recently there has been an increasing interest in TTF derivatives containing N atoms in their periphery which, while retaining the electroactive behaviour of TTF, could also be able to coordinate to transition metals [3–11]. Some TTF derivatives symmetrically substituted with cyano groups have been reported [12], but non-symmetrically substituted have been a lot less explored with the possible exception of some preligands for dithiolene complexes [13].

Aiming at enlarging this type of electron donors we report here a new non-symmetrically substituted TTF donor with one dithiin ring and one cyanobenzene ring obtained by a cross coupling reaction. These two rings are expected to enhance the degree of π-delocalization over the molecule when compared with simple TTF, while the presence of the nitrogen atom can favour specific intermolecular interaction at the molecular periphery, either coordinating to other metals as found in other TTF type ligands or promoting weak hydrogen bonds.

## Results and Discussion

### Synthesis and structure of CNB-EDT-TTF

**Scheme 1:**
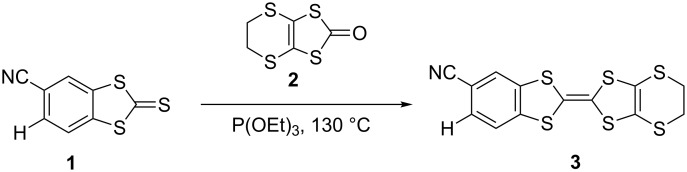
Synthesis of cyanobenzene-ethylenedithio-tetrathiafulvalene (CNB-EDT-TTF) **3**.

The synthesis of CNB-EDT-TTF **3** was obtained under a general route to prepare non-symmetrically substituted TTF derivatives by cross coupling of two different 1,3-dichalcogenole-2-chalconegones [14–15], involving the coupling between 2-thioxobenzo[*d*][1,3]dithiole-5-carbonitrile (**1**) [16] and 5,6-dihydro[1,3]dithiolo[4,5-*b*][1,4]dithiin-2-one (**2**) in 1:1.1 ratio in pure triethyl phosphite during 4 hours at 130 °C leading to the formation of **3** in relatively high yield (63%) (Scheme 1). This coupling reaction also gives rise to smaller amounts of BEDT-TTF (14% yield) and dicyanodibenzene tetrathiafulvalene (dcdb-TTF) [13] as byproducts resulting from homocoupling reactions. The separation of the products was easily achieved by chromatography. The dcdb-TTF yield was not determined since due to its insolubility it was retained at the top of the chromatography column. The yield of **3** decreased for longer reaction times and this is likely attributed to the higher formation of insoluble dcdb-TTF.

The CNB-EDT-TTF **3** electron donor is thermally stable, not sensitive to oxygen and soluble in common solvents such as CH_2_Cl_2_, CHCl_3_ and AcOEt. The molecular structure and purity of the isolated compound after column chromatography were confirmed by ^1^H and ^13^C NMR spectroscopy, UV–vis and IR spectroscopy, mass spectrometry and elemental analysis.

In the IR spectra of **3**, the characteristic C**≡**N and C=C stretching absorption bands appeared around 2229 cm^−1^ and 1637 and 1446 cm^−1^, respectively. The ^1^H NMR spectra showed signals at 7.48–7.31 and 3.33 ppm, integrating with ratios expected for the seven protons.

**Figure 1:**
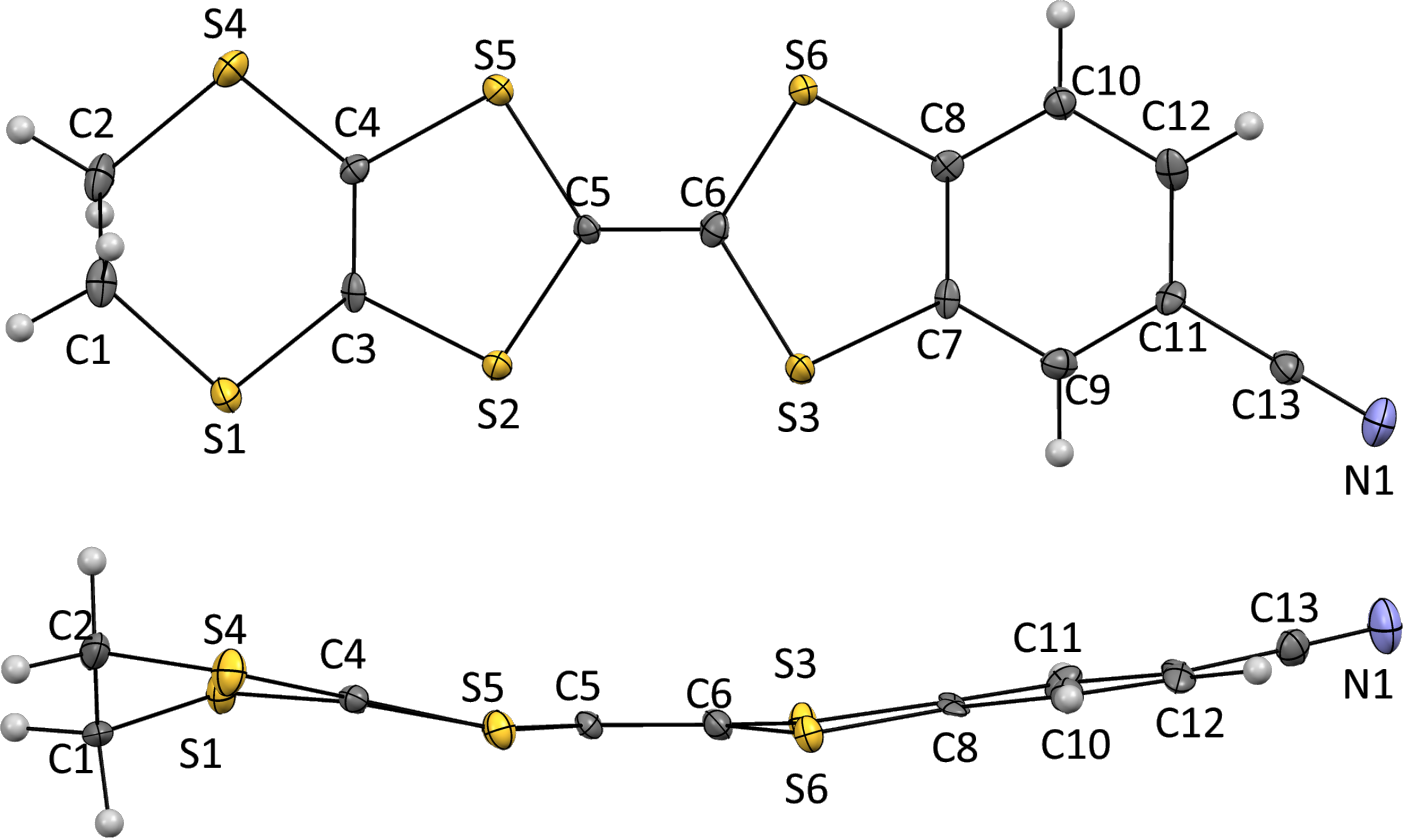
ORTEP diagrams of compound **3** drawn at 30% probability level with the atomic numbering scheme.

Single crystals of **3** suitable for X-ray analysis were grown by slow evaporation from a dichloromethane solution. The X-ray structural refinement confirm the molecular structure of the compound **3** and was found to crystallize in the monoclinic system, space group *P*2_1_/*n* with one crystallographically independent molecule in a general position (Figure 1). This molecule is almost planar; the dihydrodithiin ring adopts a half-chair conformation with five coplanar atoms. The central C=C bond length (C5–C6 = 1.355(8) Å) is typical of neutral TTF donors [17–20].

**Figure 2:**
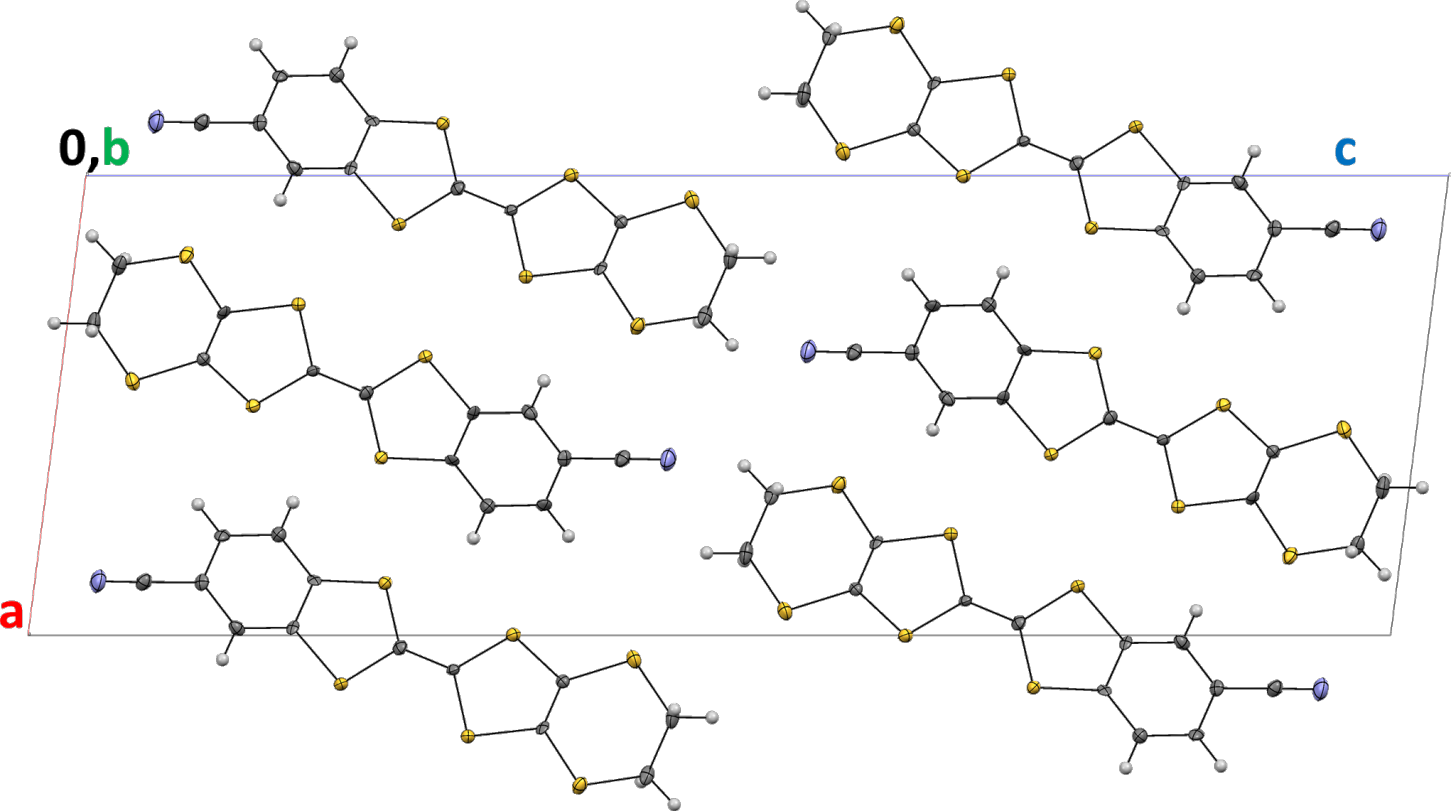
Crystal structure of compound **3** viewed along the *b* axis.

**Figure 3:**
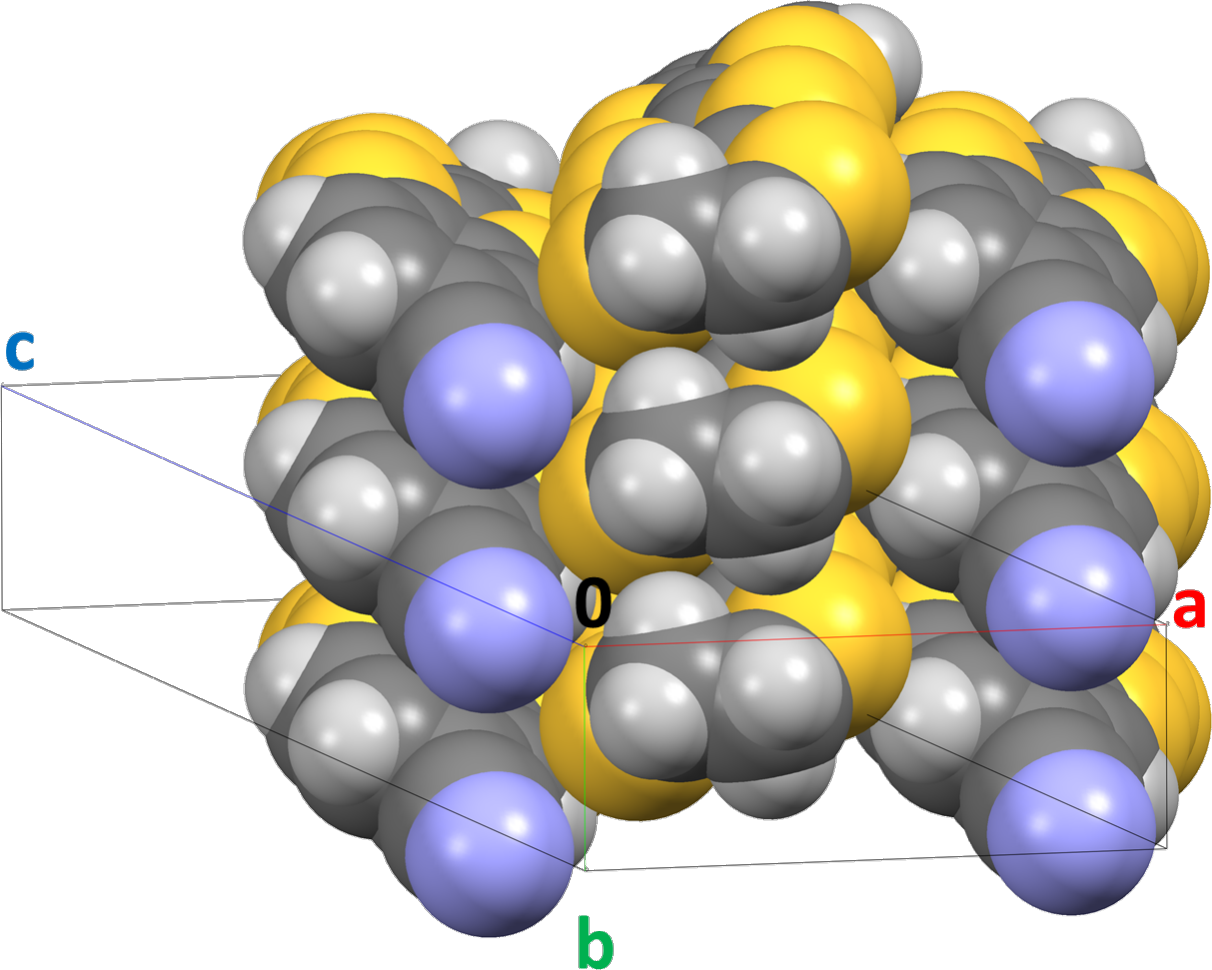
View of three neighbouring molecular stacks in **3**.

The crystal structure is made by piling up molecules head to head forming stacks along the *b* axis (Figure 2) with short S–H contacts (S1–H1B; S4–H2B) in the range 2.751–2.824 Å. Neighbouring stacks are arranged head to tail in the *a,c* plane with several short contacts between molecules in neighbouring stacks S–S, C–N, S–H, N–H (S2–S3; N1–C1; N1–H1A; S2–H9; S5–H10; S6–H10). Short-contact details are given in Table S1 in Supporting Information File 1. Molecules in neighbouring stacks along *a* are tilted by 54.35° (Figure 3).

### Spectroscopic and electrochemical properties

The electronic properties of **3** were investigated by UV–vis absorption spectroscopy in dichloromethane solution. The UV–vis spectra in DCM (Figure S6 in Supporting Information File 1) showed π–π* transitions typical of TTF donors [21], with an intense absorption band centred at approximately at 231 nm and other weaker bands at 269, 305 and 329 nm.

**Figure 4:**
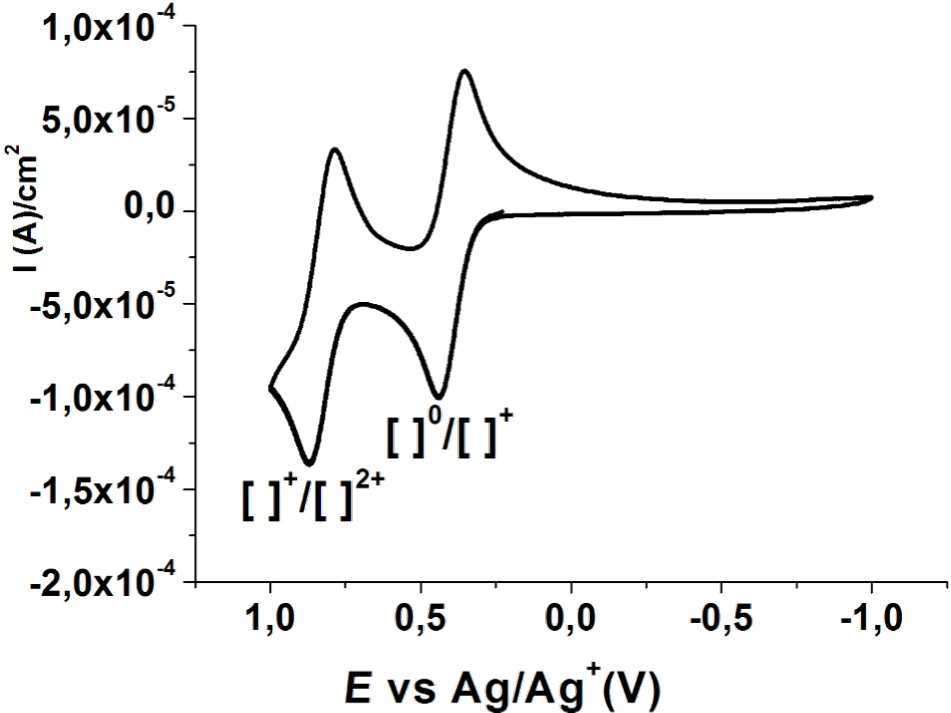
Cyclic voltammogram of **3**.

The redox properties of this donor were studied by cyclic voltammetry in dichloromethane using [*n*-Bu_4_N][PF_6_] as the supporting electrolyte (Figure 4) showing two one-electron quasi-reversible redox waves at 0.405 V and 0.850 V vs Ag/AgNO_3_ as typical of TTF donors, which are ascribed to the couples [CNB-EDT-TTF]^0^/[CNB-EDT-TTF]^+^ and [CNB-EDT-TTF]^+^/[CNB-EDT-TTF]^2+^, respectively. Comparing the redox potentials of the new TTF electron donor **3** with the well-known BEDT-TTF donor also measured by us in the same conditions, as shown in Table 1, we can conclude that, as expected, the cyanobenzene group reduces the donor properties, shifting the redox potentials to higher values due to the electron withdrawing effect of the cyano groups possibly with a partial electron transfer from the donor to the acceptor moiety. This tendency is also in agreement with the redox potentials of other cyanobenzene and pyrazine groups substituted TTFs recently reported by our group [13,22–23].

**Table 1:** Redox potentials for donors in CH_2_Cl_2_ with [*n*-Bu_4_][PF_6_] 0.1 M, *E* in V vs Ag/AgNO_3_ with scan rate = 100 mV s^−1^.

Donor	Solvent	*E*_1_^½^ (V)	*E*_2_^½^ (V)

**3**	DCM	0.405; (0.139^a^; *0.*705^b^)	0.850; (0.584^a^; 1.15^b^)
BEDT-TTF	DCM	0.234; (−0.320^a^; *0.*534^b^)	0.642; (0.376^a^; 0.942^b)^
dcdb-TTF [13]	benzonitrile	0.270	0.600
cbdc-TTF [13]	DMF	0.380	0.540

^a^vs Fc/Fc+ and ^b^vs SCE.

## Conclusion

In conclusion, we have prepared in fair yield a new non-symmetrically substituted cyanobenzene TTF-type electron donor, CNB-EDT-TTF, by a cross coupling reaction followed by chromatographic separation. The crystal structure of this donor is dominated by a large number of S-, C- and N-mediated contacts. CNB-EDT-TTF presents the electroactive behaviour of TTF-type donors although with slightly enhanced oxidation potentials due to the incorporation of the CN group. This new donor is expected to be a valuable building unit to prepare new charge transfer salts where both the dipolar moment and the possibility of metal coordination and other interactions mediated by the cyano group can be further explored to obtain compounds with interesting properties.

## Experimental

### Materials and methods

Elemental analyses of the compounds were performed using an EA 110 CE Instruments automatic analyzer. Melting points were studied on a Stuart Scientific SMP2. IR spectra were obtained on a Bruker FTIR Tensor 27 spectrophotometer. ^1^H and ^13^C NMR spectra were recorded on Brucker Avance 300 (300 MHz for ^1^H) with CDCl_3_ and (CD_3_)_2_SO used as solvents respectively and TMS the internal reference. UV–vis spectra were recorded on an UV-1800 Shimadzu spectrophotometer. Cyclic voltammetry data were obtained using a BAS C3 Cell Stand. The voltammograms were obtained at room temperature with a scan rate of 100 mV/s, platinum wire working and counter electrodes and an Ag/AgNO_3_ reference electrode. The measurements were performed on fresh solutions with a concentration of 10^−3^ M, in CH_2_Cl_2,_ that contained *n*-Bu_4_PF_6_ (10^−1^ M) as the supporting electrolyte. Mass spectra were obtained with a Bruker HCT electrospray ionization quadrupole ion trap mass spectrometer (ESI-QIT/MS). X-ray diffraction studies were performed with a Bruker APEX-II CCD detector diffractometer using graphite monochromated Mo Kα radiation (λ = 0.71073 Å), in the φ and ω scan modes. A semi empirical absorption correction was carried out using SADABS [24]. Data collection, cell refinement and data reduction were done with the SMART and SAINT programs [25]. The structures were solved by direct methods using SIR97 [26] and refined by full-matrix least-squares methods using the program SHELXL97 [27] using the winGX software package [28]. Non-hydrogen atoms were refined with anisotropic thermal parameters whereas H-atoms were placed in idealised positions and allowed to refine riding on the parent C atom. Molecular graphics were prepared using MERCURY 1.4.2 [29].

#### General procedure for the synthesis of **3**

In freshly destilled triethyl phosphite (10 mL), 2-thioxobenzo[*d*][1,3]dithiole-5-carbonitrile (**1**, 1 mmol, 0.21 g) and 5,6-dihydro[1,3]dithiolo[4,5-*b*][1,4]dithiin-2-one (**2**, 1.1 mmol, 0.23 g) were heated up to 130 °C under N_2_ at reflux for 4 h, which led to a formation of an orange precipitate. The precipitate was filtered and washed with cold methanol and dried under vacuum. The product was isolated by silica gel column chromatography with DCM:hexane (3:1) as eluent. Yield 63%; mp 238.6 °C; anal. calcd for C_13_H_7_NS_6_: C, 42.25; H, 1.91; N, 3.79; S, 52.05; found: C, 41.41; H, 2.45; N, 3.43; S, 49.25; MS *m*/*z* (100%): 369.0 (M^+^,100); ^1^H NMR (CDCl_3_, ppm) 7.46 (d, *J* = 1 Hz, 1H,), 7.38 (dd, *J* = 1, 8.1 Hz, 1H,), 7.31 (d, *J* = 8.1 Hz, 1H), 3.32 (s, 4H); ^13^C NMR (CD_3_)_2_SO) 142.8, 138.1, 130.9, 126.3, 123.9, 118.7, 113.44, 109.73, 30.11; υ_max_ (KBr)/cm^−1^: 3062 (m, Ar-H), 2922 (s, CH_2_), 2229 (s, C≡N), 1637 and 1446 (m, C=C), and 582 (m, C-S); UV–vis (DCM) λ_max_ = 231 nm.

**Crystal structure data**: The crystal structure data have been deposited at the Cambridge Crystallographic Data Centre and allocated the deposition number CCDC-1049230 for **3**. These crystallographic data can be obtained free of charge at http://www.ccdc.cam.ac.uk.

Cyanobenzene-ethylenedithio-tetrathiafulvalene **3** crystallized from dichloromethane saturated solution as orange needles, C_13_H_7_NS_6_ (*M*_r_ = 369.59), crystal size 0.35 × 0.22 × 0.18 mm^3^, monoclinic, *a* = 10.8794(6) Å, *b* = 4.1490(3) Å, *c* = 31.9854(16) Å, ß = 97.185(4), *V* = 1432.44 Å^3^, *Z* = 4, ρ_calcd_ = 1.714 g cm^−3^, space group *P*2_1_/*n*.

## Supporting Information

File 1 NMR (^1^H and ^13^C), infrared spectra, UV–vis absorption spectra and short contact list of CNB-EDT-TTF.

## References

[1] Bendikov M, Wudl F, Perepichka D F (2004). Chem Rev.

[2] Yamada J, Sugimoto T (2004). TTF Chemistry Fundamentals and Applications of Tetrathiafulvalene.

[3] Lorcy D, Bellec N, Fourmigué M, Avarvari N (2009). Coord Chem Rev.

[4] Pointillart F, Golhen S, Cador O, Ouahab L (2013). Dalton Trans.

[5] Rabaça S, Almeida M (2010). Coord Chem Rev.

[6] Griffiths J-P, Brown R J, Day P, Matthews C J, Vital B, Wallis J D (2003). Tetrahedron Lett.

[7] Branzea D G, Fihey A, Cauchy T, El-Ghayoury A, Avarvari N (2012). Inorg Chem.

[8] Belhadj E, El-Ghayoury A, Ripaud E, Zorina L, Allain M, Batail P, Mazari M, Sallé M (2013). New J Chem.

[9] Dias S I G, Neves A I S, Rabaça S, Santos I C, Almeida M (2008). Eur J Inorg Chem.

[10] Biet T, Cauchy T, Avarvari N (2012). Chem – Eur J.

[11] Biet T, Avarvari N (2014). CrystEngComm.

[12] Jia C, Liu S-X, Ambrus C, Labat G, Neels A, Decurtins S (2006). Polyhedron.

[13] Rabaça S, Oliveira S, Cerdeira A C, Simão D, Santos I C, Almeida M (2014). Tetrahedron Lett.

[14] Fabre J M (2004). Chem Rev.

[15] Fabre J M, Giral L, Dupart E, Coulon C, Manceau J P, Delhaes P (1983). J Chem Soc, Chem Commun.

[16] Cerdeira A C, Afonso M L, Santos I C, Pereira L C J, Coutinho J T, Rabaça S, Simão D, Henriques R T, Almeida M (2012). Polyhedron.

[17] Bouguessa S, Gouasmia A K, Golhen S, Ouahab L, Fabre J M (2003). Tetrahedron Lett.

[18] Liu S-X, Dolder S, Rusanov E B, Stoeckli-Evans H, Decurtins S (2003). C R Chim.

[19] Devic T, Avarvari N, Batail P (2004). Chem – Eur J.

[20] Belo D, Figueira M J, Nunes J P M, Santos I C, Almeida M, Crivillers N, Rovira C (2007). Inorg Chim Acta.

[21] Camerel F, Jeannin O, Yzambart G, Fabre B, Lorcy D, Fourmigué M (2013). New J Chem.

[22] Rabaça S, Oliveira S, Santos I C, Almeida M (2013). Tetrahedron Lett.

[23] Rabaça S, Cerdeira A C, Neves A I S, Dias S I G, Mézière C, Santos I C, Pereira L C J, Fourmigué M, Henriques R T, Almeida M (2009). Polyhedron.

[R24] 24Sheldrick, G. M. SADABS; Bruker AXS: Madison, Wisconsin, USA, 2004.

[R25] 25Bruker SMART and SAINT; Bruker AXS: Madison, Wisconsin, USA, 2004.

[26] Altomare A, Burla M C, Camalli M, Cascarano G L, Giacovazzo C, Guagliardi A, Moliterni A G G, Polidori G, Spagna R (1999). J Appl Crystallogr.

[R27] 27Sheldrick, G. M. SHELXL97, Programs for Crystal Structure Analysis, Release 97-2; Universitat Goettingen, Germany, 2008.

[28] Farrugia L J (1999). J Appl Crystallogr.

[29] Macrae C F, Edgington P R, McCabe P, Pidcock E, Shields G P, Taylor R, Towler M, van de Streek J (2006). J Appl Crystallogr.

