# Supplementary material for: Synthesis and characterization of the cyanobenzene-ethylenedithio-TTF donor
Source: Beilstein J Org Chem. 2015 Jun 3;11:951–6. doi: 10.3762/bjoc.11.106 (PMC4464436; doi:10.3762/bjoc.11.106)
Supplement: File 1 — NMR (1H and 13C), infrared spectra, UV–vis absorption spectra and short contact list of CNB-EDT-TTF. [file Beilstein_J_Org_Chem-11-951-s001.pdf]

Supporting Information

for

Synthesis and characterization of the cyanobenzene-ethylenedithio-TTF donor

Sandrina Oliveira, Dulce Belo, Isabel C. Santos, Sandra Rabaça* and Manuel Almeida*

Address: C[2]TN, Instituto Superior Técnico, Universidade de Lisboa, Estrada Nacional 10, P-2695-066 Bobadela LRS, Portugal

Email: Sandra Rabaça* - sandrar@ctn.ist.utl.pt;

Manuel Almeida* - malmeida@ctn.ist.utl.pt

*Corresponding author

NMR (^{1}H and ^{13}C), infrared spectra, UV–vis absorption spectra and short contact list of CNB-EDT-TTF

NMR spectra (^{1}H and ^{13}C) of CNB-EDT-TTF

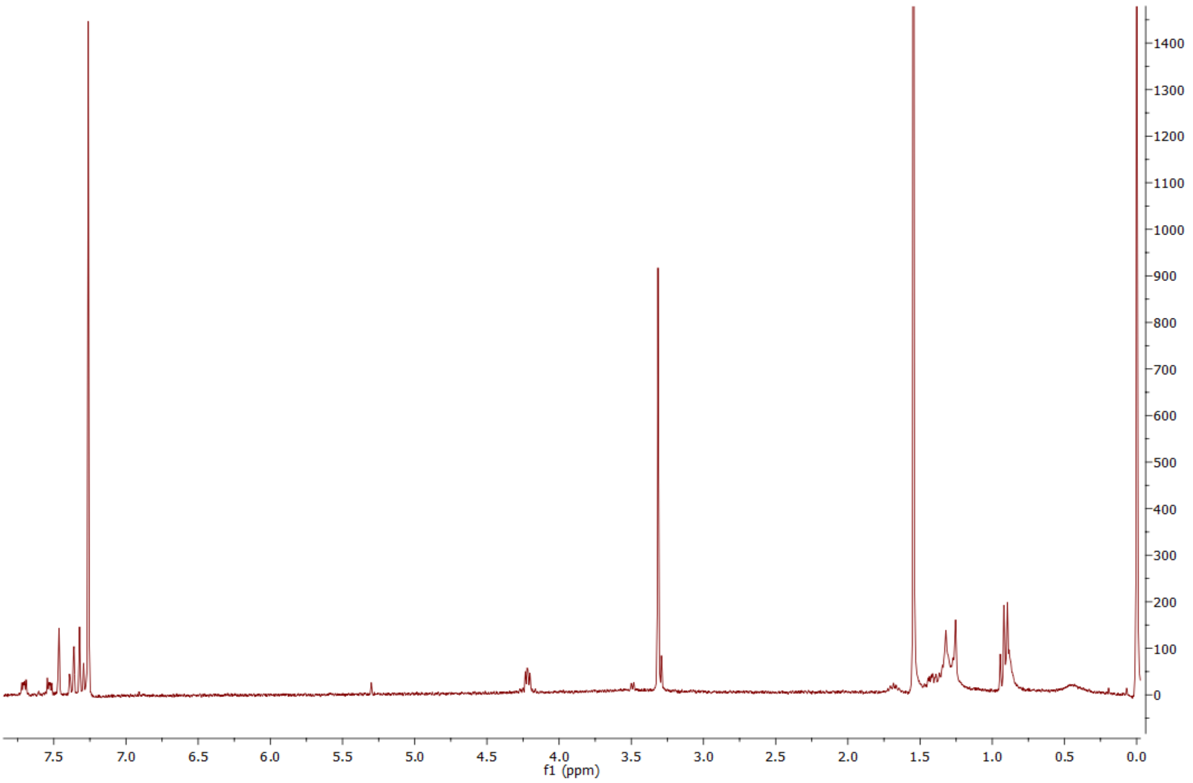

Figure S1: ^{1}H NMR spectra of compound **3**.

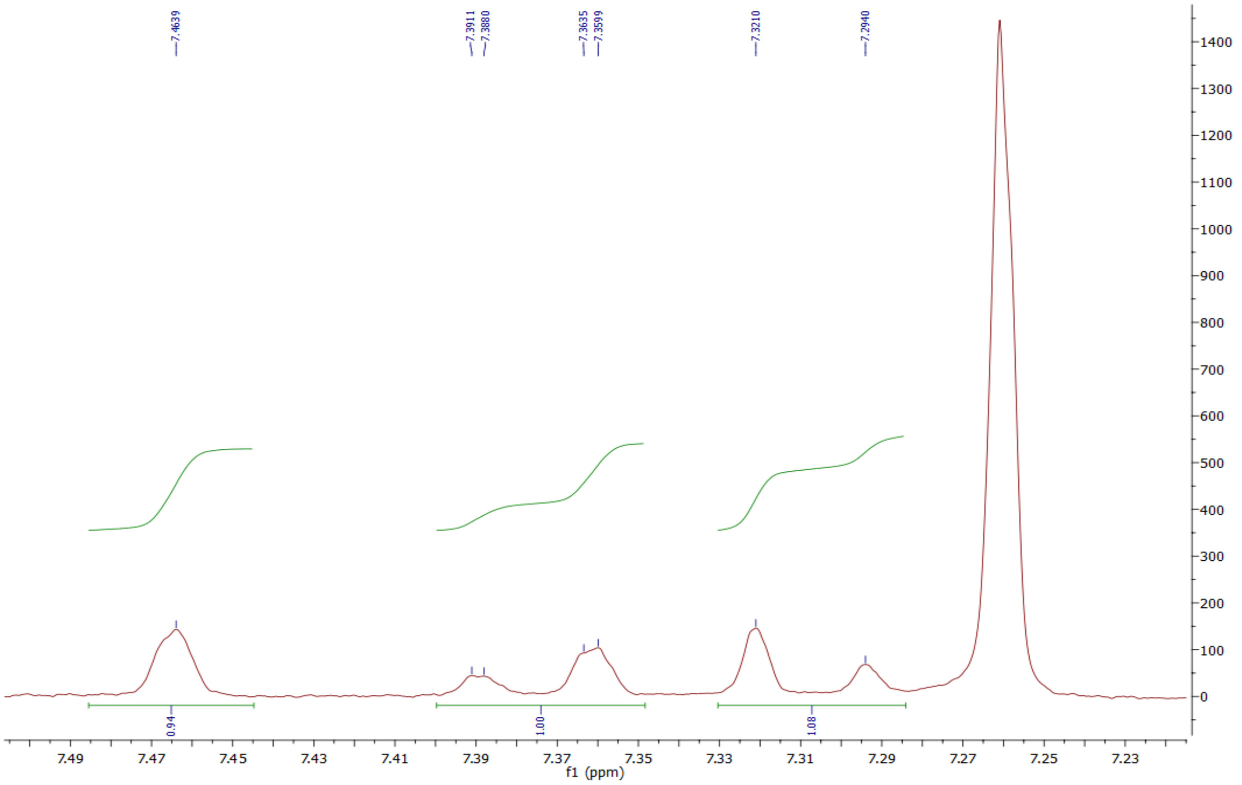

Figure S2: Zoom between 7.5 and 7.2 ppm.

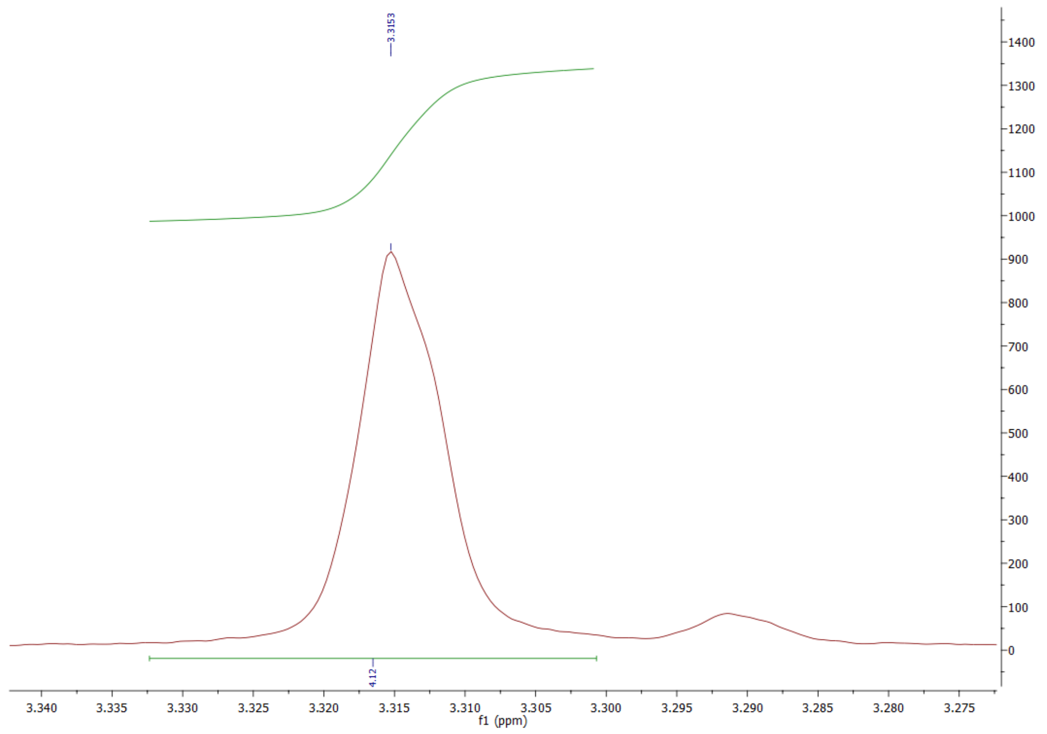

Figure S3: Zoom between 3.34 and 3.27 ppm.

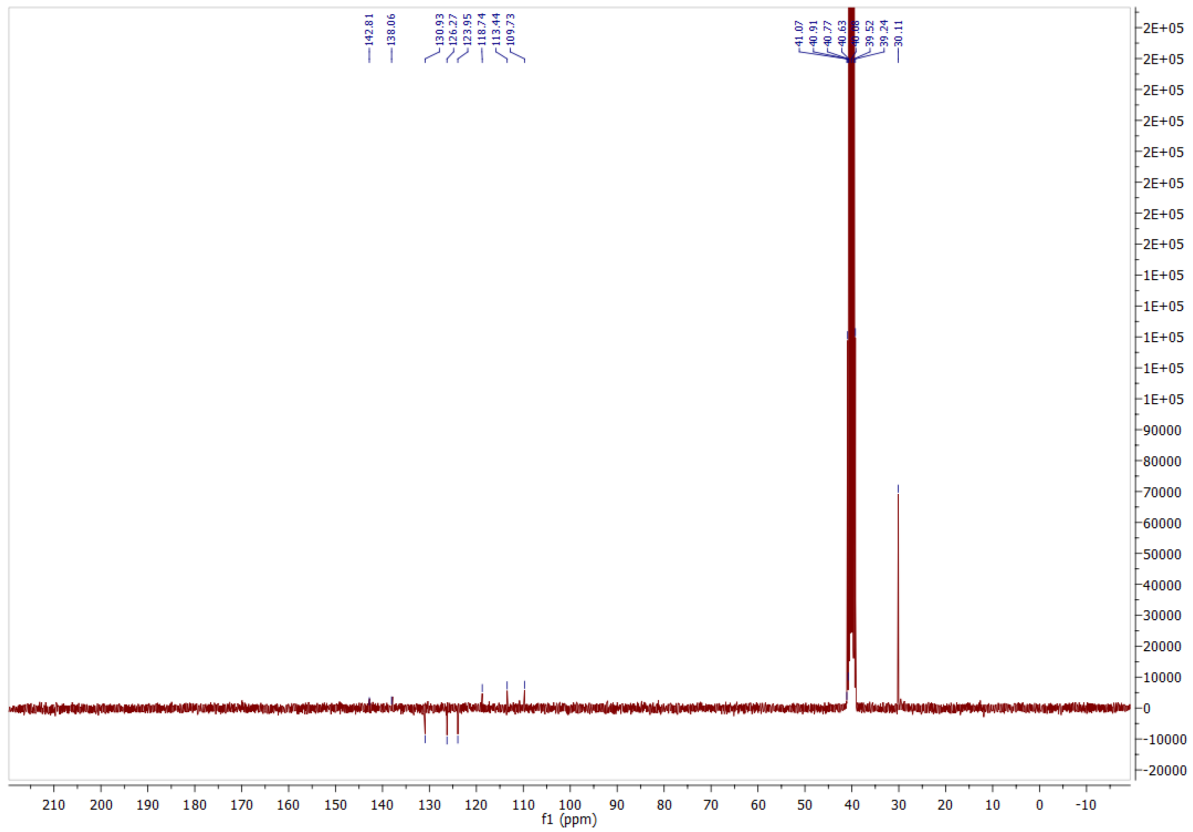

Figure S4: ^{13}C NMR spectra APT of compound **3.** APT spectra is presented “upside-down” with CH and CH_3 peaks “down” and quaternary and CH_2 peaks “up”.

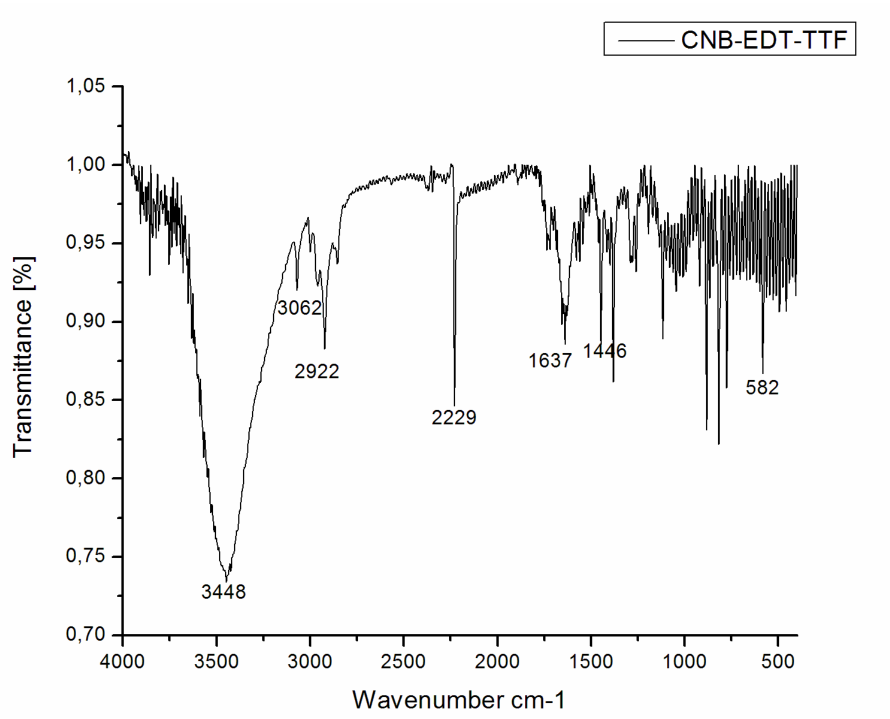

Figure S5: Infrared spectra of compound **3**.

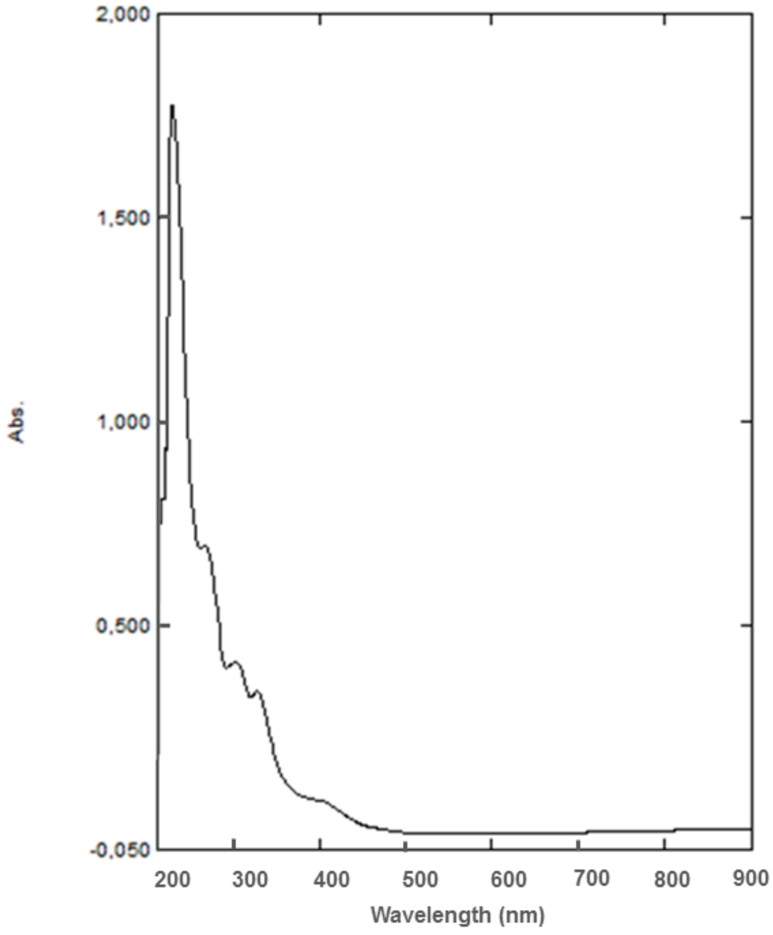

Figure S6: UV–vis absorption spectra in DCM of compound **3**.

Table S1: Short Contact list of compound **3**

Short contact	Symmetry operation	Length (Å)	Length-ΣVdW (Å)
S1⋯H1B	x,-1+y,z	2.751	-0.249
H2B⋯S4	x,-1+y,z	2.824	-0.176
S4⋯H2B	x,1+y,z	2.824	-0.176
H1B⋯S1	x,1+y,z	2.751	-0.249
S2⋯S3	1/2-x,-1/2+y,1/2-z	3.596	-0.004
H9⋯S2	1/2-x,-1/2+y,1/2-z	2.911	-0.089
S2⋯H9	1/2-x,1/2+y,1/2-z	2.911	-0.089
S3⋯S2	1/2-x,1/2+y,1/2-z	3.596	-0.004
S5⋯H10	1.5-x,-1/2+y,1/2-z	2.956	-0.044
S6⋯H10	1.5-x,-1/2+y,1/2-z	2.980	-0.020
H10⋯S5	1.5-x,1/2+y,1/2-z	2.956	-0.044
H10⋯S6	1.5-x,1/2+y,1/2-z	2.980	-0.020
H1A⋯N1	-1/2+x,1/2-y,-1/2+z	2.590	-0.160
C1⋯N1	-1/2+x,1.5-y,-1/2+z	3.231	-0.019
N1⋯H1A	1/2+x,1/2-y,1/2+z	2.590	-0.160
N1⋯C1	1/2+x,1.5-y,1/2+z	3.231	-0.019
